# Multi-locus sequence typing of *Ehrlichia ruminantium *strains from geographically diverse origins and collected in *Amblyomma variegatum *from Uganda

**DOI:** 10.1186/1756-3305-4-137

**Published:** 2011-07-15

**Authors:** Ryo Nakao, Joseph W Magona, Lijia Zhou, Frans Jongejan, Chihiro Sugimoto

**Affiliations:** 1 Department of Collaboration and Education, Research Center for Zoonosis Control, Hokkaido University, Kita 20, Nishi 10, Kita-ku, Sapporo, Hokkaido 001-0020, Japan; 2 National Livestock Resources Research Institute (NaLIRRI), P.O. Box 96, Tororo, Uganda; 3 Utrecht Centre for Tick-borne Diseases (UCTD), Department of Infectious Diseases and Immunology, Faculty of Veterinary Medicine, Utrecht University, Yalelaan 1, 3584CL, Utrecht, The Netherlands; 4 Department of Veterinary Tropical Diseases, Faculty of Veterinary Science, University of Pretoria, Private Bag X04, Onderstepoort 0110, South Africa

## Abstract

**Background:**

The rickettsial bacterium *Ehrlichia ruminantium *is the causative agent of heartwater in ruminants. A better understanding of the population genetics of its different strains is, however, needed for the development of novel diagnostic tools, therapeutics and prevention strategies. Specifically, the development of effective vaccination policies relies on the proper genotyping and characterisation of field isolates. Although multi-locus sequence typing (MLST) has been recently developed, only strains from geographically restricted collections have been analysed so far. The expansion of the MLST database to include global strains with different geographic origins is therefore essential. In this study, we used a panel of reference strains from geographically diverse origins and field samples of *E. ruminantium *detected from its vector, *Amblyomma variegatum*, in heartwater-endemic areas in Uganda.

**Results:**

A total of 31 novel alleles (six, four, six, three, two, five, three, and two for *gltA, groEL, lepA, lipA, lipB, secY, sodB*, and *sucA *loci, respectively) and 19 novel sequence types (STs) were identified. Both neighbour-joining and minimum spanning tree analyses indicated a high degree of genetic heterogeneity among these strains. No association was observed between genotypes and geographic origins, except for four STs from West African countries. When we performed six different tests for recombination (GeneConv, Bootscan, MaxChi, Chimaera, SiScan, and 3Seq) on concatenated sequences, four possible recombination events were identified in six different STs. All the recombination breakpoints were located near gene borders, indicating the occurrence of intergenic recombination. All four STs that localized to a distinct group in clustering analysis showed evidence of identical recombination events, suggesting that recombination may play a significant role in the diversification of *E. ruminantium*.

**Conclusions:**

The compilation of MLST data set across the African continent will be particularly valuable for the understanding of the existing genetic diversity of field isolates in African countries. Comprehensive information on the degree of cross-protection between strains and further understanding of possible relationships between genotypes and phenotypes such as vaccine efficacy are expected to lead to the development of region-specific vaccination strategies.

## Background

The rickettsial bacterium *Ehrlichia ruminantium *is the causative agent of heartwater in ruminants, a potential zoonotic disease [1,2] transmitted by ticks of the genus *Amblyomma *that causes considerable livestock losses in endemic countries [3]. Heartwater is distributed in nearly all countries of sub-Saharan Africa and has also extended into some islands of the Caribbean, from where it may spread into the American mainland [4]. Evidence from several vaccine trials indicate that a wide range of *E. ruminantium *genotypes with differing cross-protection capacities were simultaneously circulating in the same region [5,6], leading to a poor vaccine efficacy. Therefore, the proper genotyping and characterisation of field isolates of *E. ruminantium *is an important prerequisite for the development of effective vaccination strategies at regional levels.

Several methods have been developed to genotype *E. ruminantium*. Specifically, typing based on the *map1 *(major antigenic protein 1) gene has been extensively used and proven to be useful for estimating the genetic diversity of *E. ruminantium *strains [7-9]. However, these methods are not reliable without proper knowledge of phylogenetic relatedness. Multi-locus sequence typing (MLST) is in turn a powerful typing method that allows determining genetic diversity as well as phylogenetic relationships. Recently, Adakal et al. developed a MLST scheme for *E. ruminantium *based on eight different housekeeping genes [10]. This method was further evaluated by the same authors and proved to have a resolution high enough to discriminate even between closely related genotypes circulating in Burkina Faso [11]. However, currently available MLST profiles are limited to geographically restricted collections. Considering the wide distribution of *E. ruminantium *across the African continent, the expansion of the MLST database to include global strains from different geographic origins is therefore needed.

The aim of this study was to examine the MLST method by using a panel of reference strains from geographically diverse origins. Additionally, eight *E. ruminantium*-positive *Amblyomma variegatum *collected in Uganda were also investigated to determine the usefulness of this method for the detection of genotypes presently circulating in heartwater-endemic areas. The collection of these data sets is aimed at contributing further to the development of a global database of *E. ruminantium *genotypes.

## Methods

### *E. ruminantium *reference strains

The following 14 *E. ruminantium *strains were sequenced: Ball 3, Burkina Faso, Crystal Springs, Ifé Nigeria, Kerr Seringe, Kiswani, Kwanyanga, Lutale, Pokoase 417, Sankat 430, São Tomé, Senegal, Um Banein, and Zeerust. Their geographic origins and years of isolation are shown in Table 1. All strains were cultured in bovine aorta endothelial cells as described previously [12] and subjected to DNA extraction using the Nucleospin Tissue Kit (Macherey-Nagel, Duren, Germany).

**Table 1:** MLST profiles of *E. ruminantium *strains from geographically diverse origins and collected in *A. variegatum *from Uganda

Strain	Origin	Year of isolation	Allele							
			
			*gltA*	*groEL*	*lepA*	*lipA*	*lipB*	*secY*	*sodB*	*sucA*
Ball 3	South Africa	NR^a^	5	6	13	1	5	4	1	9
Burkina Faso	Burkina Faso	1997	1	3 (4)^b^	1	1	12	1	1	1
Crystal Springs	Zimbabwe	1990	6	7	14	6	13	15	9	9
Gardel	Guadeloupe, Caribbean	1982	1	1	3	1	4	1	1	2
Ifé Nigeria	Nigeria	1983	7	3 (4)	2	1	1	1	1	1
Kerr Seringe	The Gambia	2001	3	5	1	3	6 (7)^c^	16	3	5
Kiswani	Kenya	1985	1	8	3	1	2	17	10	4
Kwanyanga	South Africa	NR	8	3 (4)	15	7	5	15	11	9
Lutale	Zambia	1986	2	6	3	1	5	17	1	4
Pokoase 417	Ghana	1996	9	5	3	1	4	1	3	2
Sankat 430	Ghana	1996	3	5	1	3	6 (7)	16	3	5
São Tomé	São Tomé and Principe	1981	1	6	16	8	3	18	1	1
Senegal	Senegal	1981	9	5	1	3	6 (7)	19	3	5
Um Banein	Sudan	1981	2	6	17	1	5	17	1	4
Welgevonden (Erwe)	South Africa	1981	2	3 (4)	2	1	5	1	1	4
Welgevonden (Erwo)	South Africa	1985	2	3 (4)	2	1	5	4	1	4
Zeerust	South Africa	1979	10	6	18	1	13	15	1	10
*A. variegatum *samples from Uganda										
A004	Amuria, Uganda	2008-2009	1	9	17	1	1	1	1	4
A006	Amuria, Uganda	2008-2009	1	9	17	1	1	1	1	4
D002	Dokolo, Uganda	2008-2009	2	3 (4)	3	1	8	17	1	4
P003	Pallisa, Uganda	2008-2009	1	3 (4)	17	1	3	17	1	4
P006	Pallisa, Uganda	2008-2009	2	3 (4)	3	1	8	17	1	4
S001	Soroti, Uganda	2008-2009	2	3 (4)	3	1	8	1	1	4
S013	Soroti, Uganda	2008-2009	1	6	2	1	1	1	1	1
T009	Tororo, Uganda	2008-2009	1	9	17	1	1	1	1	4

No. of alleles			9	7	9	5	9	7	5	6
No. of mutated sites per locus			15	11	10	11	13	24	7	12
Size (bp)			395	447	475	341	358	587	415	401
Polymorphism (%)			3.80	2.46	2.11	3.23	3.63	4.09	1.69	2.99
DI^d^			80.3	80.0	85.7	42.0	88.7	78.3	47.0	73.7
dN/dS^e^			0.36	0.22	0.43	0.22	0.30	0.14	0.40	0.33

^a^NR, not previously recorded in the literature.

^b^The sequences of STs 3 and 4 of *groEL *deposited in GenBank are identical.

^c^The sequences of STs 6 and 7 of *lipB *deposited in GanBank are identical.

^d^DI, Simpson's diversity index.

^e^dN/dS, the ratio of nonsynonymous to synonymous substitutions.

### *E. ruminantium*-positive *A. variegatum *from Uganda

Eight *E. ruminantium*-positive tick samples detected by pCS20 PCR [13] were used. Ticks were collected from indigenous cattle from December, 2008 to January, 2009 at heartwater-endemic areas in five different districts in Uganda: Amuria (33.38°W, 02.01°N), Dokolo (33.10°W, 01.55°N), Pallisa (33.42°W, 01.10°N), Soroti (33.36°W, 01.43°N), and Tororo (34.11°W, 00.41°N). A total of two (A004 and A006), one (D002), two (P003 and P006), two (S001 and S013), and one (T009) samples were collected in Amuria, Dokolo, Pallisa, Soroti, and Tororo, respectively.

### Sequencing analysis

The following eight housekeeping genes were used for DNA sequencing: *gltA, groEL, lepA, lipA, lipB, secY, sodB*, and *sucA*. PCR amplification was conducted using the KAPA 2G Robust PCR Kit as reported by Adakal et al. [10]. PCR products were purified with ExoSAP-IT (USB Corporation, Cleveland, OH) and sequenced using the BigDye Terminator version 3.1 Cycle Sequencing Kit (Applied Biosystems, Foster City, CA) and an ABI Prism 3130 x genetic analyzer (Applied Biosystems) according to the manufacturer's instructions. The DNA sequences obtained were submitted to the DNA Data Bank of Japan (DDBJ) (http://www.ddbj.nig.ac.jp) under accession numbers AB625780 to AB625787 (*gltA*), AB625788 to AB625796 (*groEL*), AB625797 to AB625810 (*lepA*), AB625811 to AB625824 (*lipA*), AB625825 to AB625838 (*lipB*), AB625839 to AB625852 (*secY*), AB625853 to AB625860 (*sodB*), and AB625861 to AB625874 (*sucA*).

### Data analysis

Sequences were analysed using GENETYX version 9.1 (GENETYX Corporation, Tokyo, Japan) and were trimmed on both the 5' and 3' ends according to the previous report [10]. The Gardel, Welgevonden (Erwe), and Welgevonden (Erwo) sequences were obtained from the GenBank database (GenBank accession numbers: CR925677, CR925678, and CR767821, respectively). Eight genotypes (Strains 2, 331, 469, 623, 629, 630, 668, and 1062) identified in *A. variegatum *from Burkina Faso [10] were subjected to phylogenetic, cluster, and recombination analyses. A phylogenetic analysis was carried out for concatenated sequences of eight genes using MEGA 4 programme [14]. Allele sequences for each strain were concatenated in the order *gltA*-*sucA*-*lepA*-*sodB*-*lipA*-*secY*-*lipB*-*groEL *resulting in a final composite length of 3,419 bp. The phylogenetic tree was constructed using the neighbour-joining (NJ) method with 1,000 bootstrap replicates. For each MLST locus, an allele number was assigned to each unique variant. Those sequences identical to previously known alleles were assigned the same allele numbers, whereas those that did not match any known allele were given new allele numbers. A sequence type (ST) was assigned to each distinct combination of alleles at the eight MLST loci. A minimum-spanning tree (MST) was generated using BioNumerics software version 6.5 (Applied Maths, Saint-Martens-Latem, Belgium). The Simpson's diversity index (DI) was calculated for each locus to determine its discriminatory power. Alignment files of each gene locus and concatenated sequences of all loci were imported into SplitsTree4 program version 4.11.3 [15] and a preliminary network was obtained using the neighbour-net algorithm [16]. PHI test was conducted to determine whether recombination events were statistically significant. We further evaluated recombination events using six methods implemented in Recombination Detection Program version 3.44 (RDP3) [17], including GeneConv [18], Bootscan [19], MaxChi [20], Chimaera [21], SiScan [22], and 3Seq [23]. If one sequence was identical to another sequence, only one representative sequence from each group was included in this analysis.

## Results and Discussion

All MLST loci were successfully amplified from the 14 reference strains and eight Ugandan tick samples. The sequencing analysis of the amplified PCR products revealed that 103 nucleotide sites (3.01%) were polymorphic in a total of 3,419 bp from the eight MLST loci (Table 1). The lowest number of polymorphisms per locus was seven in the *sodB*, followed by 10 in *lepA*, 11 in the *groEL *and *lipA*, 12 in the *sucA*, 13 in the *lipB*, 15 in the *gltA*, and 24 in the *secY *locus. The percentage of polymorphic sites ranged from 1.69% in *sodB *to 4.09% in the *secY *locus. Since multiple sequences were not detected by direct sequencing of PCR products, we assumed that the sequences obtained from each locus originated from a single population.

*SodB *was the most conserved locus among the strains examined in this study, as similarly found for a previous comparison among *E. ruminantium*-positive tick samples in Burkina Faso [11], indicating that *sodB*, and specifically *E. ruminantium*-specific *sodB *LAMP [13], is a suitable target for the genetic identification of this species. Conversely, the locus with highest percentage of polymorphic sites was *secY *(4.09%), which is similar to the percentage previously observed for Burkina Faso (4.60%) [11]. Out of 103 single nucleotide polymorphisms, a total of 22 (21.4%) resulted in nonsynonymous amino acid changes. The ratios of nonsynonymous to synonymous substitutions (dN/dS) were 0.36, 0.22, 0.43, 0.22, 0.30, 0.14, 0.40, and 0.33 for *gltA, groEL, lepA, lipA, lipB, secY, sodB*, and *sucA *loci, respectively, indicating an accumulation of synonymous mutations at all loci.

Three sets of strains or tick samples, the pair Kerr Seringe and Sankat 430, the pair D002 and P006, and the triplet A004, A006, and T009, had identical sequences in all eight MLST loci. Although Kerr Seringe and Sankat 430 were from geographically isolated countries (The Gambia and Ghana, respectively [24,25]), a high level of similarity in the sequences from these strains have been previously reported based on the analysis of the highly polymorphic *map1 *gene [8]. Therefore, using only these target genes might not be sufficient to enable the discrimination between closely related strains.

Allele numbers were assigned to each sequence as indicated in Table 1. A total of 31 novel alleles were identified (six, four, six, three, two, five, three, and two for *gltA, groEL, lepA, lipA, lipB, secY, sodB*, and *sucA *loci, respectively). To evaluate the discriminatory power of each locus, DI values were calculated using BioNumerics software. DI ranges from 0 to 100, namely from a low to a high diversity. The lowest DIs were associated with the *lipA *(42.0) and *sodB *(47.0) loci, suggesting that these genes are the least informative, as the DI values for the other loci were higher than 70.0. This result is also reflected in the identical allelic profiles for the *lipA *and *sod B *loci in the eight Ugandan tick samples (Table 1). STs were determined for each reference strain and tick sample based on the alleles identified at each locus. Out of 25 samples (17 reference strains and eight Ugandan tick samples), a total of 21 STs were identified. Except for the STs of the Gardel and Welgevonden (Erwo) strains, which were also included in a previous study [10], 19 STs were novel.

**Figure 1:**
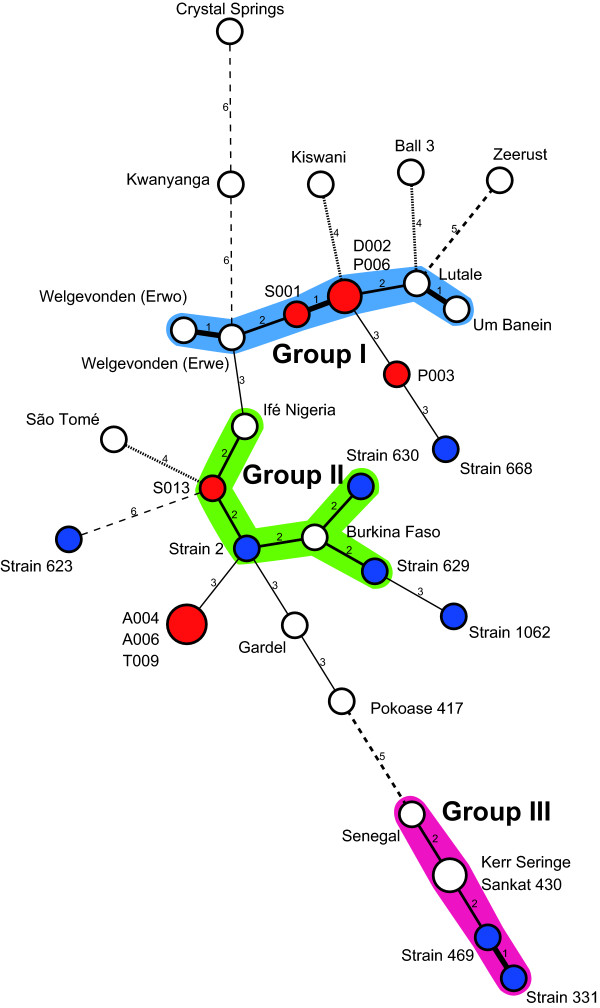
**Minimum-spanning tree based on MLST profiles**. Each circle in the tree represents a different ST. The white, red, and blue circles represent reference strains, *A. variegatum *samples from Uganda, and *A. variegatum *samples from Burkina Faso, respectively. Circle size is proportional to the numbers of strains or tick samples belonging to an ST. Numbers between circles represent the number of allelic differences. Two or more STs differing at less than two loci are regarded as a group and are distinguished by a different colour. The strength of the link (bold, plain, or discontinuous) indicates the degree of genetic similarity (number of common alleles) between STs.

In addition to 17 reference strains and eight Ugandan samples, eight STs previously detected in *A. variegatum *from Burkina Faso were included for further analyses. We first performed a cluster analysis based on ST profiles. The resulting MST revealed the existence of three main groups (named I, II, and III) (Figure 1). Group I was the largest, consisting of four reference strains and three Ugandan tick samples. Group II was composed of two reference strains and three samples from Burkina Faso and one sample from Uganda. Group III consisted of three reference strains and two samples from Burkina Faso. Eight reference strains and seven samples, three from Burkina Faso and four from Uganda, were not included in any group. There was no association between groups and geographic origins, except for four STs in group III, all of which originated from West African countries (Burkina Faso, Ghana, Senegal, and The Gambia). A phylogenetic analysis based on a 3,419-bp concatenated sequence of eight genes revealed that these four STs were also clustered together, while others were not clustered according to their geographic origins in a NJ tree (Additional file 1). Therefore, even though MST and NJ analyses rely on distinct analytical principles, both methods suggested a high degree of genetic heterogeneity among the strains examined and highlighted the genetic isolation of STs in group III.

There are several hypotheses that could explain the cause of a lack of association between STs and their geographic origins. For example, the migration of *E. ruminantium *from other endemic regions, presumably due to transportation of infected animals or ticks, as previously evidenced by the introduction of this pathogen into the Caribbean [26], could be responsible. Another possible cause is the temporal spread in terms of the year of isolation; for example, the Ball 3 strain was isolated prior to 1952 [27], while field samples from Burkina Faso and Uganda were collected in 2007-2008 [10] and 2008-2009, respectively. However, we cannot rule out the possibility that this typing method, particularly when employed with the current set of target loci, is not suitable for tracing geographic origins of bacteria, for example, because of the effect of recombination between different genotypes.

**Figure 2:**
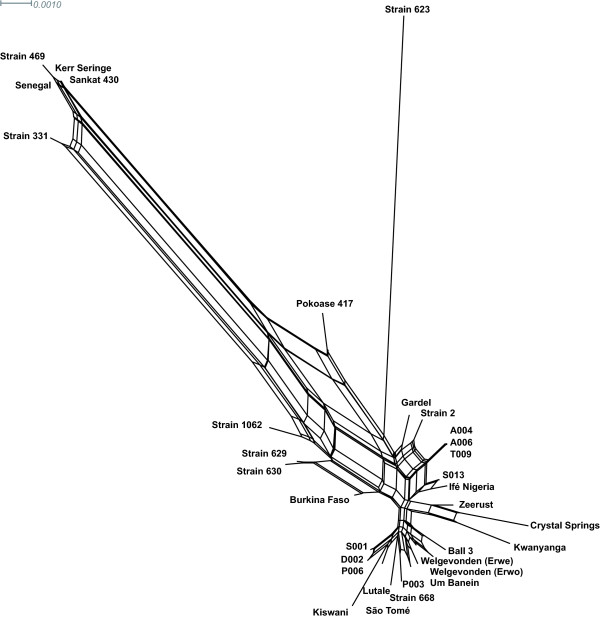
**Neighbour-net network constructed from concatenated sequences obtained from all eight loci**. The eight housekeeping genes were concatenated in the order *gltA*-*sucA*-*lepA*-*sodB*-*lipA*-*secY*-*lipB*-*groEL*. Significant evidence of recombination was obtained by the PHI test (*P *= 0.0).

We next conducted a neighbour-net analysis to examine the impact of recombination on each locus separately and on the concatenated sequence of all STs. The resulting graphs based on the alignments of individual loci showed tree-like structures and the PHI test did not detect evidence of intragenic recombination (Additional file 2). However, the graph obtained from an alignment of concatenated sequences showed a network structure (Figure 2), providing the evidence for the genetic divergence among the STs examined and highlighting the influence of recombination events on the evolution of this bacterial species (PHI test on the significance of recombination: *p *= 0.0).

**Figure 3:**
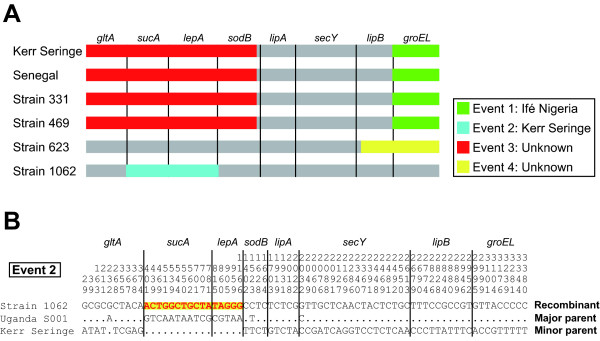
**Detection of recombination events**. (A) Schematic representation of four recombination events identified by RDP3. Each gray bar represents a concatenated sequence of a recombinant. Colour bars indicate the insertion of foreign sequences. The event numbers and the putative origins of foreign sequences are shown in the box. The gene order of the concatenated sequence is shown on the top and the borders are shown as vertical lines. (B) Mosaic structure in recombination event 2. The alignment was conducted on the concatenated sequences of one recombinant (Strain 1062) and two parental STs (Kerr Seringe and Uganda S001). Only the polymorphic sites are shown, and the position on the sequence is shown above each site. A foreign sequence is indicated as red on yellow. The gene order of the concatenated sequence is shown on the top and the borders are shown as vertical lines.

In order to find further evidence for intergenic recombination, we performed six different tests for recombination on concatenated sequences. Four possible recombination events were identified in six different STs (Kerr Seringe/Sankat 430, Senegal, Strains 331, 469, 623, and 1062) (Table 2). A schematic representation of the events is shown in Figure 3A. Events 1 and 2 were supported by all six tests, while events 3 and 4 were supported by three different tests. All the recombination breakpoints were located near gene borders, indicating intergenic recombination.

**Table 2:** The detection of putative recombination events using six different tests

Event	Breakpoint		Recombinant	Major parent	Minor parent	Detection method					
					
	Beginning	Ending				GeneConv	Bootscan	MaxChi	Chimaera	SiScan	3Seq
1	2965	3419	Kerr Seringe^a ^ Senegal Strain 331 Strain 469	Unknown	Ifé Nigeria	+	+	+	+	+	+
2	388	1284	Strain 1062	S001	Kerr Seringe	+	+	+	+	+	+
3	1	2651	Kerr Seringe Senegal Strain 331 Strain 469	Strain 630	Unknown	+				+	+
4	2652	3419	Strain 623	A004^b^	Unknown			+		+	+

^a^A representative ST of Kerr Seringe and Sankat 430.

^b^A representative ST of A004, A006, and T009.

+, detection of significant recombination (*p  *< 0.05).

Strain 1062 from Burkina Faso was predicted to be the result of recombination between S001 from Uganda as a major parent and Kerr Seringe from The Gambia (or Sankat 430 from Ghana) as a minor parent (Table 2). When their sequences were aligned, a clear mosaic pattern was observed (Figure 3B). In addition, A004 (or A006/T009) from Uganda was predicted to be a major parent of a putative recombinant, Strain 623 from Burkina Faso. Considering the geographical isolation between major and minor parents of these recombinants (Strains 623 and 1062), this result may support the idea of the existence of a homogeneous population of an ancestral genotype throughout the African continent as previously hypothesised by Adakal et al. [11].

All STs clustered in group III by MST analysis showed evidence of identical recombination events (events 1 and 3) (Table 2 and Figure 3A), indicating that recombination may play a significant role in the diversification of *E. ruminantium *as previously suggested [10,28-30]. In this collection of samples, however, all the STs predicted to be recombinants originated from West African countries. One possible explanation for this regional restriction is that the recombination events could not be properly detected, for example, due to the biased sampling and/or low levels of genetic diversity among the tested strains. Therefore, further compilation of the MLST data, especially of currently circulating isolates in East and Southern African countries, will be invaluable for understanding the role of recombination in bacterial genome evolution and for providing an overview of the current situation of bacterial genetic diversity in African countries.

Finally, the recombination events identified in this study demonstrate that a multi-locus genotyping approach, rather than single-gene based genotyping, is a prerequisite for a proper understanding of phylogenetic relationships of *E. ruminantium*. The failure to discriminate between two closely related strains, Kerr Seringe and Sankat 430, highlights the need to either improve the MLST method or to develop other multi-locus genotyping methods with higher resolution power, such as the multi-locus variable-number tandem repeat analysis.

## Conclusions

We investigated a recently developed MLST scheme that allows direct genotyping of *E. ruminantium *by using global strains from diverse origins and field samples from heartwater-endemic areas in Uganda. As only a limited dataset consisting of geographically restricted isolates was available from previous reports, this study expands the number of allele variants known for each locus. The analyses presented here also provide strong evidence for the occurrence of recombination events among the STs examined. The compilation of MLST data across the African continent will be particularly valuable for understanding the existing genetic diversity of field isolates in African countries. Comprehensive information on the degree of cross-protection between strains and further understanding of possible relationships between genotypes and phenotypes such as vaccine efficacy are expected to lead to the development of region-specific vaccination strategies.

## Competing interests

The authors declare that they have no competing interests.

## Authors' contributions

RN performed PCR and sequencing, conducted data analysis, and draft the manuscript. JWM carried out tick sampling in Uganda. LZ conducted DNA extractions, PCR and sequencing. CS and JF conceived of the study, and participated in its design and coordination and helped to finalize the manuscript. All authors read and approved the final manuscript.

## Supplementary Material

Additional file 1 **Neighbour-joining phylogenetic tree based on concatenated sequences obtained from all eight loci**. The tree was constructed based on a 3,419-bp concatenated sequence of eight housekeeping genes. One thousand bootstrap replicates were performed for each analysis. Bootstrap values are shown at the nodes. Click here for file

Additional file 2 Split graph constructed from the sequences of each locus. Click here for file
