# Supplementary material for: Multi-locus sequence typing of Ehrlichia ruminantium strains from geographically diverse origins and collected in Amblyomma variegatum from Uganda
Source: Parasit Vectors. 2011 Jul 15;4:137. doi: 10.1186/1756-3305-4-137 (PMC3151223; doi:10.1186/1756-3305-4-137)

## Additional file 1. Neighbour-joining phylogenetic tree based on concatenated sequences obtained from all eight loci.

The tree was constructed based on a 3,419-bp concatenated sequence of eight housekeeping genes. One thousand bootstrap replicates were performed for each analysis. Bootstrap values are shown at the nodes.


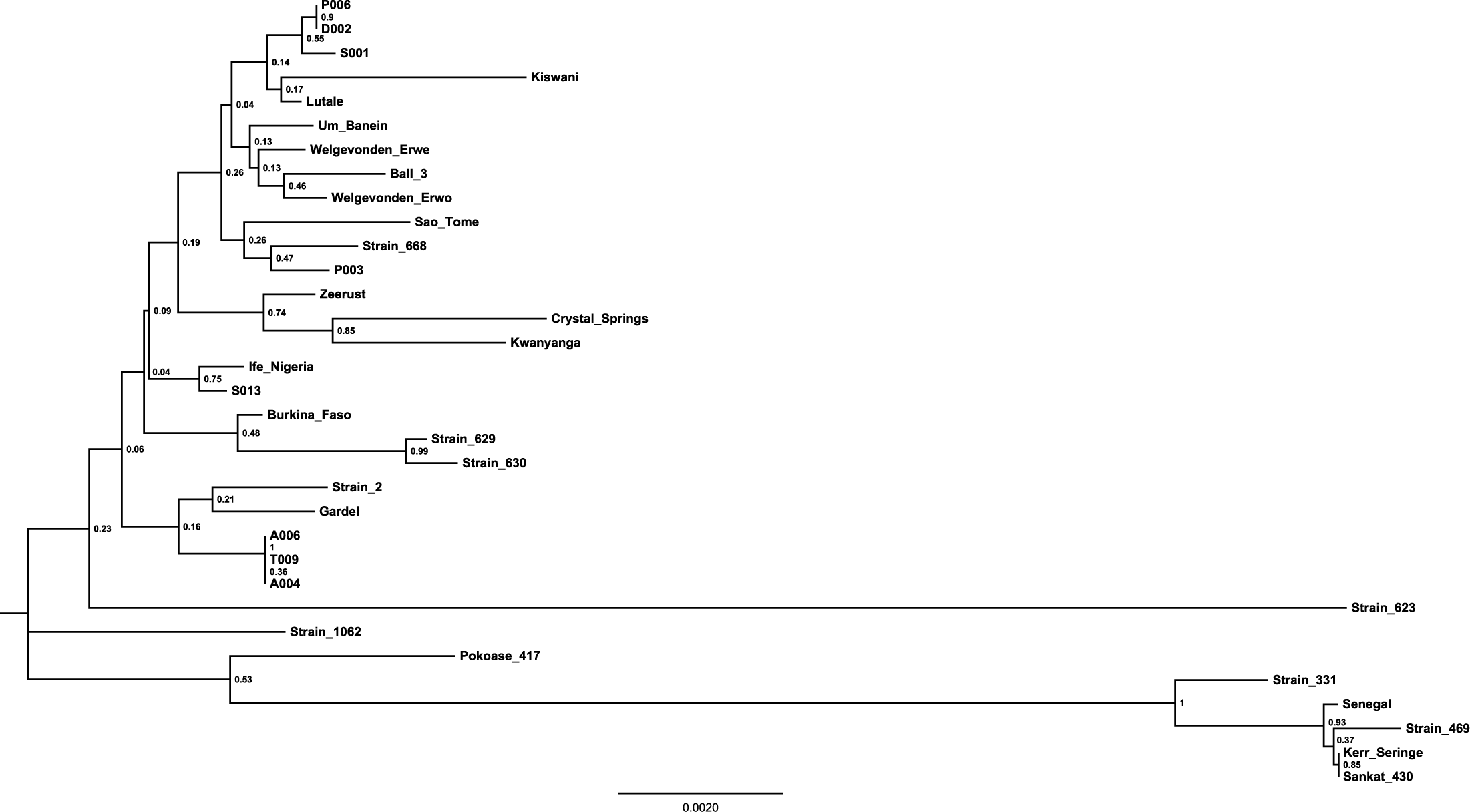

Supplement: Additional file 1 — Neighbour-joining phylogenetic tree based on concatenated sequences obtained from all eight loci. The tree was constructed based on a 3,419-bp concatenated sequence of eight housekeeping genes. One thousand bootstrap replicates were performed for each analysis. Bootstrap values are shown at the nodes. [file 1756-3305-4-137-S1.DOC]
