# Supplementary figures and images for: Multi-locus sequence typing of Ehrlichia ruminantium strains from geographically diverse origins and collected in Amblyomma variegatum from Uganda
Source: Parasit Vectors. 2011 Jul 15;4:137. doi: 10.1186/1756-3305-4-137 (PMC3151223; doi:10.1186/1756-3305-4-137)

## Additional file 2. Split graph constructed from the sequences of each locus.


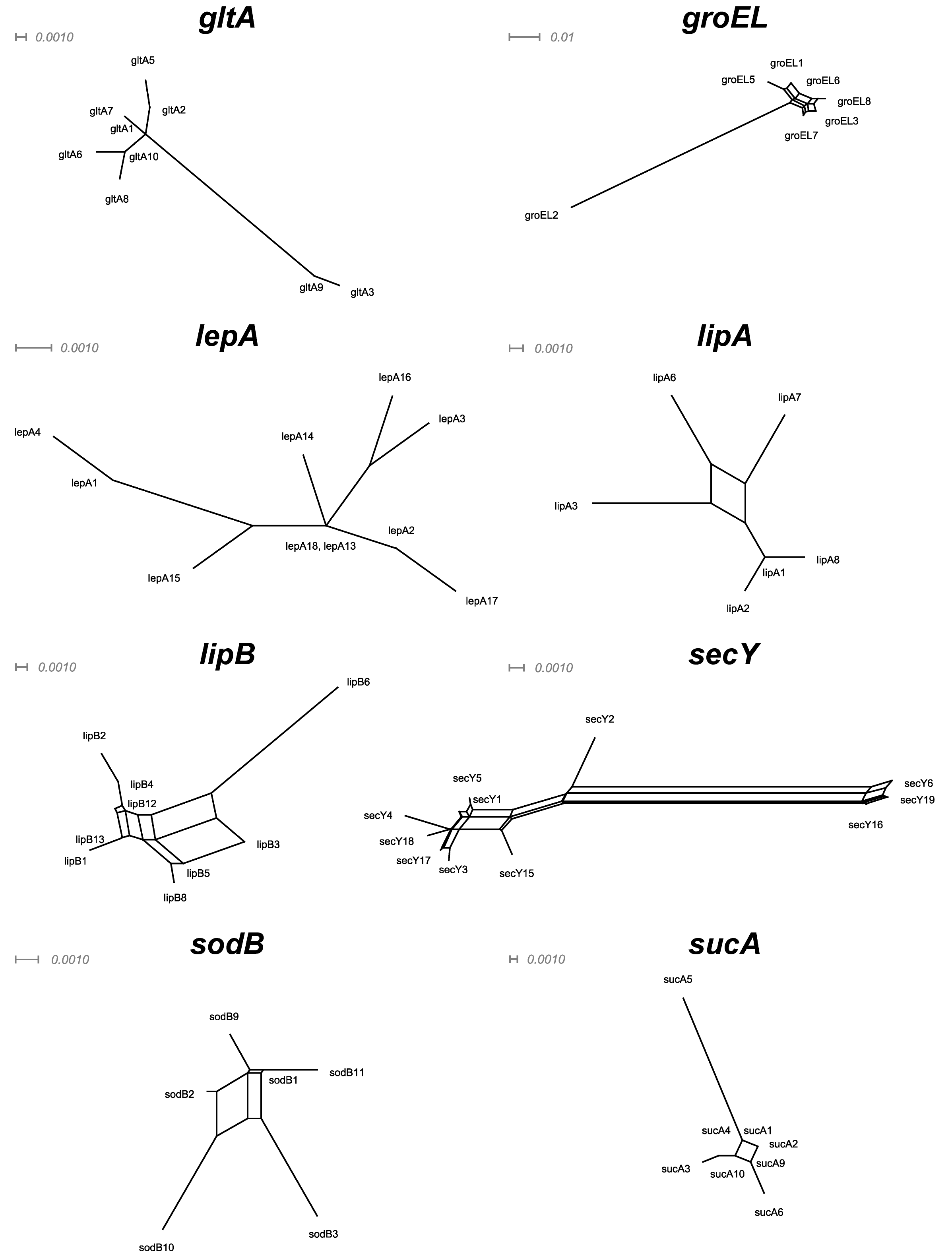

Supplement: Additional file 2 — Split graph constructed from the sequences of each locus. [file 1756-3305-4-137-S2.DOC]
